# Development and application of an integrated allele-specific pipeline for methylomic and epigenomic analysis (MEA)

**DOI:** 10.1186/s12864-018-4835-2

**Published:** 2018-06-15

**Authors:** Julien Richard Albert, Tasuku Koike, Hamid Younesy, Richard Thompson, Aaron B. Bogutz, Mohammad M. Karimi, Matthew C. Lorincz

**Affiliations:** 1 Department of Medical Genetics, The University of British Columbia, Vancouver, BC Canada; 2 Department of BioScience, Tokyo University of Agriculture, Setagaya-ku, Tokyo, Japan; 3 Graphics Usability and Visualization Lab, School of Computing Science, Simon Fraser University, Burnaby, BC Canada; 4 Canada’s Michael Smith Genome Sciences Centre, British Columbia Cancer Agency, Vancouver, BC Canada; 5 Biomedical Research Centre, The University of British Columbia, Vancouver, BC Canada; 6 Qatar Biomedical Research Institute, Hamad Bin Khalifa University, Doha, Qatar; 7 MRC London Institute of Medical Sciences, Imperial College, London, UK

**Keywords:** Epigenomics, Allele-specific, Allelic, RNA-seq, Chromatin immunoprecipitation, ChIP, ChIP-seq, Whole genome bisulphite-sequencing, WGBS, Imprinting, MEA

## Abstract

**Background:**

Allele-specific transcriptional regulation, including of imprinted genes, is essential for normal mammalian development. While the regulatory regions controlling imprinted genes are associated with DNA methylation (DNAme) and specific histone modifications, the interplay between transcription and these epigenetic marks at allelic resolution is typically not investigated genome-wide due to a lack of bioinformatic packages that can process and integrate multiple epigenomic datasets with allelic resolution. In addition, existing ad-hoc software only consider SNVs for allele-specific read discovery. This limitation omits potentially informative INDELs, which constitute about one fifth of the number of SNVs in mice, and introduces a systematic reference bias in allele-specific analyses.

**Results:**

Here, we describe MEA, an INDEL-aware Methylomic and Epigenomic Allele-specific analysis pipeline which enables user-friendly data exploration, visualization and interpretation of allelic imbalance. Applying MEA to mouse embryonic datasets yields robust allele-specific DNAme maps and low reference bias. We validate allele-specific DNAme at known differentially methylated regions and show that automated integration of such methylation data with RNA- and ChIP-seq datasets yields an intuitive, multidimensional view of allelic gene regulation. MEA uncovers numerous novel dynamically methylated loci, highlighting the sensitivity of our pipeline. Furthermore, processing and visualization of epigenomic datasets from human brain reveals the expected allele-specific enrichment of H3K27ac and DNAme at imprinted as well as novel monoallelically expressed genes, highlighting MEA’s utility for integrating human datasets of distinct provenance for genome-wide analysis of allelic phenomena.

**Conclusions:**

Our novel pipeline for standardized allele-specific processing and visualization of disparate epigenomic and methylomic datasets enables rapid analysis and navigation with allelic resolution. MEA is freely available as a Docker container at https://github.com/julienrichardalbert/MEA.

**Electronic supplementary material:**

The online version of this article (10.1186/s12864-018-4835-2) contains supplementary material, which is available to authorized users.

## Background

Next-generation sequencing (NGS)-based approaches for genome-wide analysis of RNA, histone post-translational modifications (PTMs), DNA methylation (DNAme) and chromatin conformation are now routinely conducted on both model organisms and human samples. Such studies have yielded many insights into the interplay between chromatin structure and transcription, including the surprising observation that allele-specific phenomena may be more widespread than previously believed [1, 2]. Unfortunately, while such datasets, including RNA sequencing (RNA-seq), chromatin immunoprecipitation followed by sequencing (ChIP-seq) and whole genome bisulphite sequencing (WGBS), are theoretically amenable to allele-specific profiling, NGS analysis software generally does not discriminate between parental alleles from diploid genomes. Indeed, popular read aligners depend on alignment to a single reference genome, essentially considering the sequencing reads generated from autosomes (and the X-chromosome in the case of females) as originating from isogenic rather than outbred individuals. In merging both parental alleles into a single measurement, these aligners neglect allele-specific phenomena, such as genomic imprinting [1], X-chromosome inactivation [2] and sequence-dependent *cis*-regulatory effects [3].

To overcome this shortcoming, a number of software packages have recently been developed that assign NGS sequencing reads to a specific parental allele. For example, MMSEQ [4], QuASAR [5], MBASED [6] and SCALE [7] were designed to analyze RNA-seq data, while MethPipe [8], epiG [9] and BSPAT [10] were designed to process DNAme data. Several independent custom scripts for allele-specific analyses have also been reported [11–13], but the details required for implementing them were not included. Pipelines such as Allelome.PRO [14], WASP [15] and our previously published toolbox, ALEA [16] accommodate both RNA- and ChIP-seq datasets, yet no pipeline offers the additional capability of processing DNAme data. The lack of a universal allele-specific pipeline has precluded robust integration of allele-specific transcription, histone PTMs and DNAme profiles. Importantly, while such pipelines can be applied in parallel to analyze distinct epigenomic features, installation and implementation of multiple software packages can be time consuming, even for experienced bioinformaticians. Additionally, comparing allelic results generated using different software can introduce confounding factors, as the strategies used to process reads depend on multiple parameters, including read trimming, alignment mismatch scoring and read alignment filtering (mapping quality, PCR duplicate reads). For example, several allele-specific analysis packages rely on reference genome alignment followed by variant calling [8, 10, 14], while others leverage publicly available single nucleotide variant (SNV) data to derive a diploid genome for read alignment [5, 15, 16]. This “pseudogenome” strategy is a significant improvement over the former as it enables alignment over loci with high levels of genetic variation. However, current pipelines exclude short insertions and deletions (INDELs) for pseudogenome reconstruction, as they modify reference chromosome sequence lengths and annotated gene coordinates required for downstream analyses. Given the relative abundance of INDELs, this shortcoming may lead to the omission of a significant fraction of informative allelic reads. Indeed, analysis of high quality genotyping information for mouse strains reveals that, exclusive of structural variants, INDELs compose up to 20% of genetic variation [17]. Thus, an INDEL-aware allele-specific pipeline that considers both SNVs and INDELs for pseudogenome reconstruction would offer a significant improvement over existing software.

**Fig. 1:**
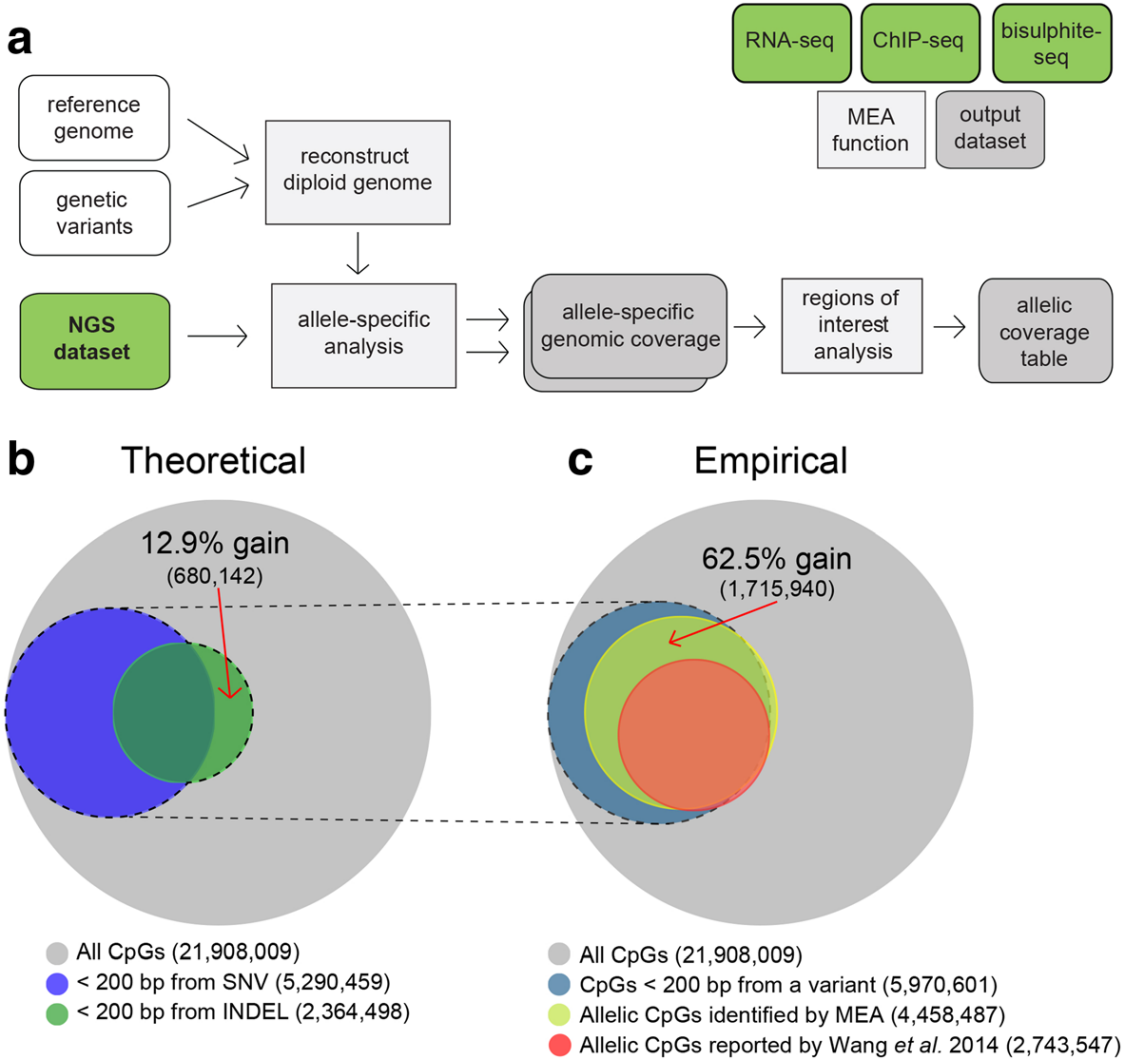
A bioinformatics toolkit for allele-specific epigenomic analysis. **a** MEA pipeline flow chart. Supplied with a reference genome assembly and relevant genetic variants, MEA first reconstructs a diploid pseudogenome. Subsequently, allele-specific analysis is performed on the input gene expression (RNA-seq), histone PTM (ChIP-seq) or DNAme (WGBS) data in FASTQ format. MEA calculates allelic imbalance values using the resulting allele-specific genomic coverage files and generates a tab-delimited table for the user-defined regions of interest. Mouse and human exon, gene body and transcription start site coordinates are provided to facilitate analyses of such regions. **b** Venn diagram showing the theoretical number of CpG dinucleotides for which allele-specific DNAme levels can be calculated using C57BL/6 J and DBA/2 J SNVs (blue) or INDELs (green) alone. CpGs for which allelic information can theoretically be extracted are defined as those that fall within 200 bp (an insert size typical of WGBS libraries) of a genetic variant. **c** Venn diagram showing the observed number of C57BL/6 J-specific CpG dinucleotides for which allele-specific DNAme levels were calculated using MEA (yellow) versus an INDEL-agnostic contemporary allele-specific DNAme script [11] using the same dataset (red)

Here, we present MEA, an “all-in-one” bioinformatics toolbox that exploits both SNVs and INDELs to enable allele-specific analyses of RNA-seq and ChIP-seq as well as WGBS datasets generated using short-read sequencing technology **(**Fig. 1a**)**. MEA is shipped in a Docker container, enabling one step installation of all dependencies independent of operating system type. After providing a reference genome assembly (e.g. hg19 or mm10) and a VCF file containing the relevant genetic variants, users simply input an NGS dataset in FASTQ format. MEA will then automatically generate allele-specific genomic coverage files in BigWig format and allele-specific analyses over user-defined regions of interest in a tab-delimited table. To benchmark the performance of our INDEL-aware software, we present both theoretical and real-world evidence for improved allele-specific DNAme analysis relative to an INDEL-agnostic pipeline. Furthermore, to highlight the utility of MEA, we investigate DNAme data processed in parallel with RNA- and ChIP-seq data from mouse hybrid embryos and uncover novel differentially methylated regions (DMRs). Additionally, using human brain cell data, we observe the expected H3K27ac and DNAme enrichment at known imprinted genes and uncover novel monoallelically expressed genes, further demonstrating the power of integrating epigenetic and expression analyses in a unified workflow. The MEA toolbox harmonizes NGS read processing, with all dependencies consolidated in a Docker container, includes pan-species compatibility, maximizing its utility for allele-specific profiling of model organisms as well as human samples.

## Implementation

To generate a harmonized workflow for processing of DNAme, RNA-seq and ChIP-seq datasets, we developed a universal strategy for detecting allele-specific reads. Further, to maximize the number of experimental reads that can be assigned to a specific allele for each data type, MEA was designed to exploit underlying genetic variation by incorporating both SNVs and INDELs during pseudogenome construction. For each data type, allelic reads are captured by constructing an in silico pseudogenome comprised of both parental genomes followed by NGS read alignment. Aligning reads simultaneously to both haplotype sequences of a diploid genome facilitates the appropriate alignment of reads that map to heterozygous loci onto their cognate allele, reads which otherwise would be discarded due to “sequencing errors”. Such reads are thus extracted and can be used to de-convolute allelic phenomena.

### An allele-specific DNA methylation pipeline

To establish a pipeline for allele-specific DNAme analysis, we began by incorporating Bismark [18], a widely adopted bisulphite-seq read aligner and methylation caller, into ALEA, our previously developed tool for allele-specific analyses of RNA-seq and ChIP-seq datasets [16]. We first quantified the hypothetical increase in the percentage of informative CpG sites from which we can infer allelic information by incorporation of INDELs in addition to SNVs during pseudogenome reconstruction. As high-quality genetic variation information of inbred mouse strains is available [19], we constructed a pseudogenome from two mouse strains, namely DBA/2 J and the reference strain C57BL/6 J (build mm10), incorporating known genetic variants (SNVs and/or INDELs). By counting CpGs within 200 bp (an insert size typical of WGBS libraries) of an INDEL or SNV, we found that INDEL incorporation leads to a theoretical increase in the number of informative CpGs (i.e. CpGs for which DNAme differences between alleles can be deduced) of 12.9% for this pseudogenome (Fig. 1b). Notably, a subset of genomic regions with associated INDELs are entirely devoid of SNVs and therefore include nearby CpGs that theoretically can only be assessed by pipelines that are “INDEL-aware”.

## Results

### MEA is informative for significantly more CpGs than an INDEL-agnostic script

To test whether the inclusion of INDELs increases the number of informative CpGs for which allelic methylation state can be calculated in practice, we processed raw reads from a previously published WGBS dataset from C57BL/6 J x DBA/2 J mouse F1 inner cell mass (ICM) cells [11]. Applying the same filtering parameters allowed us to directly compare results obtained with the MEA pipeline to those of the Bismark-based INDEL-agnostic custom script employed by Wang et al. [11]. MEA yielded a 62.5% increase in the number of CpGs covered by at least 5 allele-specific C57BL/6 J reads (Fig. 1c). Importantly, informative CpGs gained using MEA overlapped almost exclusively with CpGs within 200 bp of an INDEL or SNV, as expected. This gain is likely the result of an increase in the number of informative heterozygous sites (quantified in Fig. 1b) as well as efficacious alignment of reads to the non-reference genome over regions with high INDEL density.

**Fig. 2:**
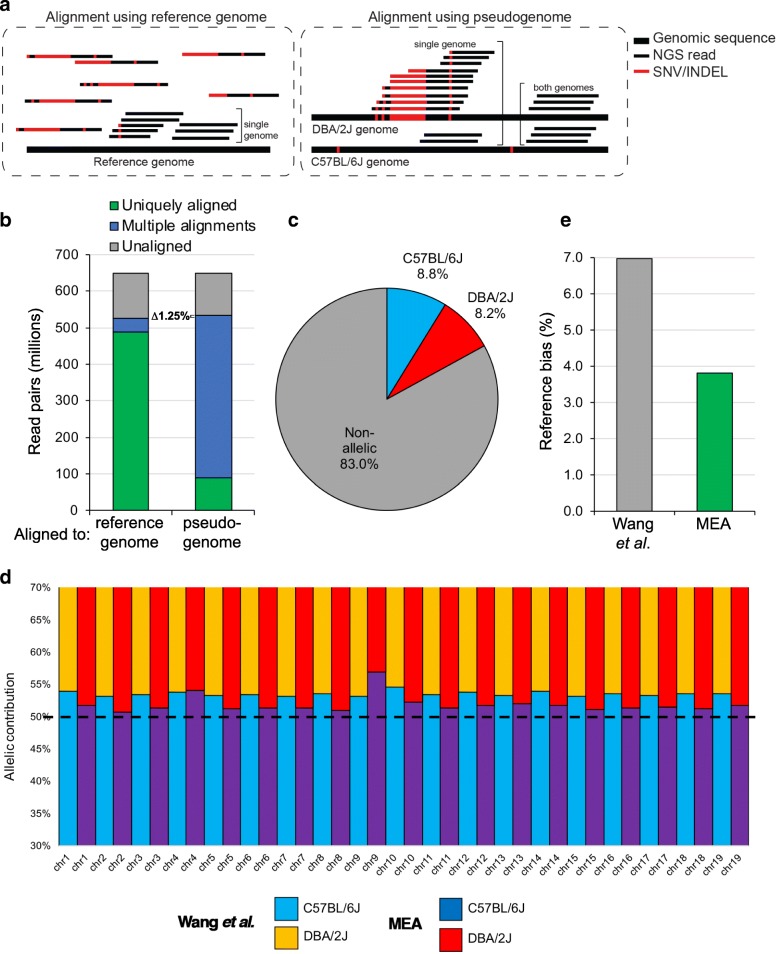
Empirical benchmarking of allele-specific read alignment reveals reduced reference bias. **a** Graphical representation of MEA’s unified strategy for detecting allele-specific reads from RNA-, ChIP-seq and WGBS datasets. Aligning F1 hybrid reads to a pseudogenome enables alignment to their cognate genome even when originating from highly variable loci. **b** Paired-end WGBS reads (101 bp) from a previously published dataset of C57BL/6 J x DBA/2 J ICM cells [11] were aligned using the Bismark aligner to the (haploid) reference genome (mm10 build) and a MEA-constructed diploid pseudogenome. When using MEA, multiple (2 or more) alignments reflect non-allelic reads, while uniquely aligned reads are allele-specific. Reads aligning uniquely to the pseudogenome were extracted and retroactively assigned to their parental haplotype. **c** The percentages of allele-specific reads called for each parental haplotype and the number of aligned reads that did not overlap with a genetic variant (non-allelic) is shown. **d** Allelic contribution of read alignments to each parental haplotype (C57BL/6 J or DBA/2 J) on each autosome. Relative to the script employed by Wang et al. [11], MEA displays about half the reference bias on the majority of autosomes. **e** Global reference bias for each pipeline is shown

Reads from regions with high INDEL density were presumably excluded by the pipeline from Wang et al. [11] as “sequencing errors”, rather than assigned as allelic variants. To confirm that MEA increases the alignment rate of non-reference reads, we repeated the alignment of C57BL/6 J x DBA/2 J F1 WGBS reads to a reference genome as well as the MEA-constructed diploid pseudogenome (composed of the reference and DBA/2 J genomes) and determined the number of reads that aligned to each genome 0, 1 or > 1 time (Fig. 2a-b). Alignment to a pseudogenome increased the overall alignment rate by 1.25% (80.83 to 82.08%), most likely due to alignment of non-reference-originating reads at loci that show significant genetic divergence (high SNV and INDEL density) from the reference. As expected, the majority of reads aligned uniquely to the haploid reference genome aligned at least twice to the pseudogenome, except over regions containing genetic variants. This crucial distinction allowed the uniquely aligned reads to be extracted and assigned to their cognate parental genomes, with 8.8 and 8.2% of all aligned reads specific to C57BL6J and DBA/2 J strains, respectively (Fig. 2c). By capturing a greater number of sites at which we can measure allelic DNAme levels, a higher proportion of experimental reads can be assigned to a specific parental haplotype, thus enabling the evaluation of allelic differences in DNAme levels for a higher fraction of the genome.

### MEA significantly reduces reference genome alignment bias

A major concern when exploring allele-specific data is the potential for reference bias caused by differences in genomic sequence quality between the reference and non-reference genomes, which may lead to preferential alignment of reads to the former and artefactual allelic imbalance results [20]. For example, using an INDEL-agnostic pipeline similar to that employed by Wang et al. [11], Keown et al*,* reported a reference bias of 15.4% in their study of allele-specific DNAme in C57BL/6 J x SPRET/EiJ cells [21] (SPRET/EiJ has > 5 times the number of SNVs relative to C57BL/6 J than does DBA/2 J [19]). To determine the extent of reference bias in our MEA pipeline, we benchmarked the observed parental contribution to allelic read alignment for each autosome from the C57BL/6 J x DBA/2 J ICM WGBS dataset generated by Wang et al. [11] **(**Fig. 2d**)**. Notably, MEA yielded an alignment reference bias on all autosomes of 3.81%, only ~ 54% of that reported by the INDEL-agnostic pipeline (6.98%, Fig. 2e). This reduction in alignment bias is consistent with the increased fraction of allele-specific reads aligned to the non-reference genome.

### Estimation of allele-specific alignment error rate using isogenic mice

**Fig. 3:**
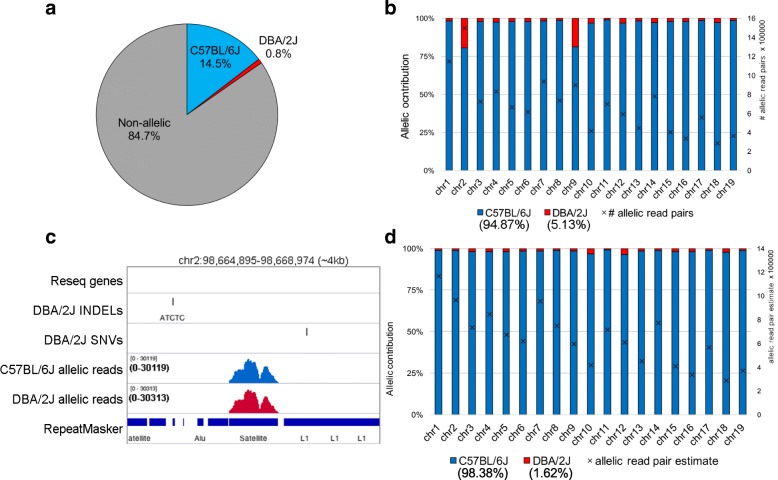
Quantifying allele-specific alignment error rates. To estimate the rate of false-positive errors for allelic analysis of DNAme data, WGBS reads generated from C57BL/6 J mice [11] were aligned to the MEA-generated C57BL/6 J x DBA/2 J pseudogenome, and the percentage of DBA/2 J-specific read alignments was scored. The expected allelic contribution from C57BL/6 J is 100%, as these cells are of C57BL/6 J origin. **a** The percentages of reads aligning uniquely to the C57BL/6 J and DBA/2 J (false-positive) pseudogenomes, as well as the number of aligned reads that did not overlap with a genetic variant (non-allelic) is shown. **b** The false-positive alignment rate for each autosome, along with the total number of aligned allelic read pairs, is shown. **c** Genome browser screenshot of a locus that displays a high rate of false-positive allele-specific alignment to a repeat annotated as Satellite DNA by RepeatMasker and devoid of genetic variants. **d** To assess the false-positive rate exclusive of repetitive Satellite DNA, allele-specific read alignments over these Repbase annotated repetitive sequences, as recognized by RepeatMasker, were culled and the rate of false-positive allele-specific alignments recalculated over each autosome as in (**b**)

False positives caused by erroneous allelic read alignment at regions devoid of true genetic variation can lead to an underestimation of reference bias in allele-specific experiments. To quantify the false positive allelic alignment rate of our pipeline, we processed pure C57BL/6 J WGBS data using the C57BL/6 J x DBA/2 J pseudogenome described above and determined the parental contribution to allelic read alignment (Fig. 3a). Curiously, 0.8% of all aligned reads (5.13% of allelic reads) were scored as DBA/2 J-specific, indicating that MEA has an FDR of ~ 5%. When calculating the parental contribution to allelic read alignment over each autosome, we found that the majority of false-positive (“DBA/2 J-specific”) allelic read alignments clustered on chromosomes 2 and 9 (Fig. 3b). Closer inspection revealed that these regions are annotated by RepeatMasker as Satellite DNA **(**Fig. 3c**)**. Such allele-specific calls at sites lacking genetic variants are the result of Bismark’s mapping quality algorithm, which calculates an erroneously high mapping score at these highly repetitive regions. Analysis of processed WGBS data from pure DBA/2 J spermatozoa without black-listing of repetitive regions revealed a C57BL/6 J-specific alignment rate of 3.80% (Additional file 1: Figure S1), indicating that a global false positive rate of ~ 5% may be expected when using the MEA pipeline for analysis of WGBS data without excluding repetitive regions. Since satellite DNA is generally omitted in studies of the transcriptome or epigenome, we excluded reads aligned to annotated satellite repeats (0.19% of the mappable genome) and recalculated the false-positive rate for the C57BL/6 J dataset, which dropped to 1.62% of allelic reads, with no specific chromosome enriched **(**Fig. 3d**)**. Thus, when applying the MEA pipeline, the majority of false positive read alignments can likely be removed by black-listing satellite repeats.

### MEA reports the expected allelic imbalance in DNA methylation at known gametic differentially methylated regions (gDMRs)

**Fig. 4:**
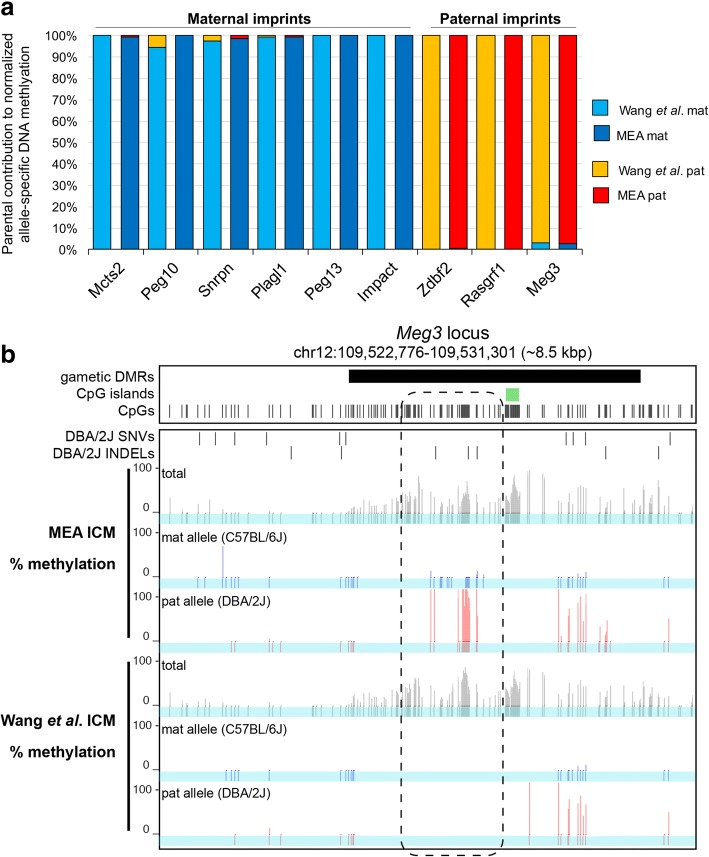
Validation of allele-specific DNA methylation level calculations over known gDMRs. C57BL/6 J x DBA/2 J ICM WGBS reads were processed in parallel with MEA and a published pipeline [11] using identical parameters. **a** Allelic methylation levels over 9 known gDMRs are shown for both pipelines. **b** UCSC genome browser screenshot of the *Meg3* gDMR including the allele-agnostic percentage of DNAme calculated using each pipeline (total) as well as allelic calls for each informative CpG. The location of each informative CpG for each pipeline (blue tracks) is also included. Only MEA detects allele-specific reads in a region within the gDMR that lacks SNVs but contains several INDELs (dashed box). A summary of the total number of allelic CpG counts and DNAme levels over this locus is included in Table 1

To establish the accuracy of calculating allele-specific DNAme levels using the MEA pipeline, we measured allele-specific DNAme levels over known imprinted gDMRs. Such regions are densely methylated on one allele and unmethylated on the other as a result of parent-of-origin dependent differences in methylation established in the gametes, representing a unique resource for benchmarking allele-specific DNAme calling. Of the 23 known mouse gDMRs, 9 harbor SNVs and/or INDELs between the C57BL/6 J and DBA/2 J genomes and can therefore be assessed for allele-specific DNAme levels. For consistency, we directly compared our allele-specific results over these regions with those reported by Wang et al. [11] **(**Fig. 4a**)**. For most gDMRs, MEA yielded average allelic DNAme levels similar to those reported by the INDEL-agnostic pipeline. However, MEA consistently yielded allele-specific information over a greater number of CpGs (mean ± SD: 72 ± 24 vs 38 ± 21 CpGs on either allele), increasing the statistical power of allelic imbalance calculations. For example, MEA detected a total of 68 CpGs informative for allelic methylation state at the *Meg3* gDMR, nearly three times greater than the number reported by Wang et al. **(**Table 1**)**. As expected, when calculated over the same 129 CpGs covered by at least five reads in the gDMR, DNAme levels calculated by the two pipelines independent of allelic calling were nearly identical (30.2% vs 30.6%). However, the discordance between the percentage of methylation calculated for the CpGs that are informative at an allelic level was significantly lower using the MEA pipeline (0.13% vs 5.8%), indicating that the accurate determination of allelic DNAme levels at specific loci can be adversely impacted by sampling errors. Furthermore, as expected, only the MEA pipeline yields informative results for CpGs proximal to INDELs at the *Meg3* gDMR locus **(**Fig. 4b**)**, confirming the benefit of incorporating the latter during pseudogenome reconstruction. Taken together, these analyses demonstrate that MEA outperforms an INDEL-agnostic pipeline.

**Table 1:** Allele-specific DNA methylation level analysis over the *Meg3* gDMR

Pipeline	Allelic call	CpGs covered	Mean Methylation (%)
MEA	–	129	30.24
	C57BL/6 J	31	1.66
	DBA/2 J	37	58.55
	Total allelic informative	68	30.11
Wang et al. (Table S7)	–	129	30.63
	C57BL/6 J	12	1.59
	DBA/2 J	12	48.09
	Total allelic informative	24	24.84

### MEA uncovers novel putative transient DMRs at annotated transcription start sites (TSSs)

**Fig. 5:**
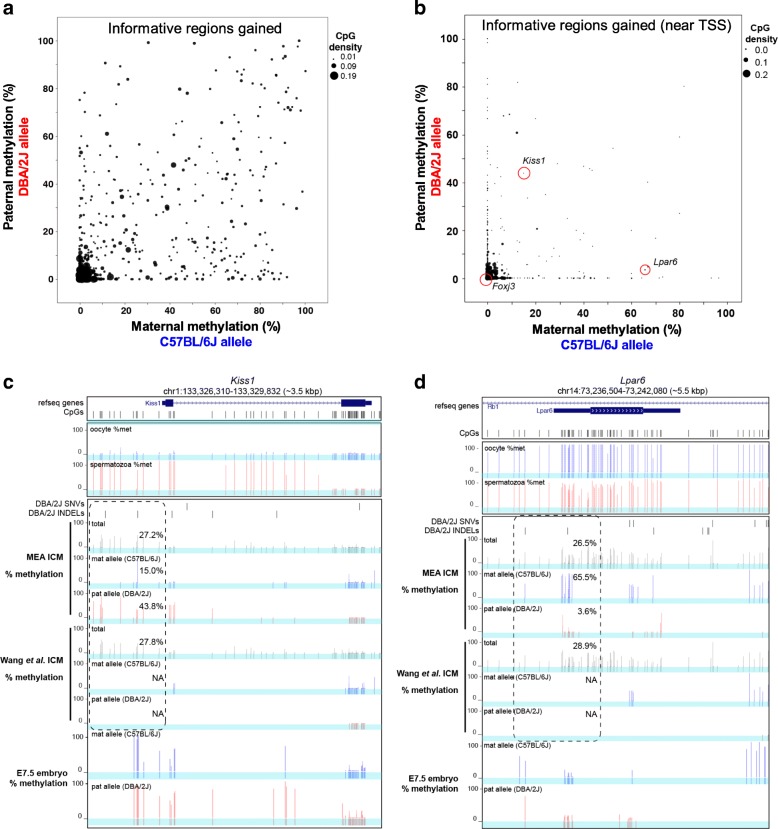
Identification of novel DMRs using the MEA pipeline. Allele-specific DNAme levels were calculated over 133,065 regions containing INDELs but lacking SNVs (representing novel informative regions gained employing MEA) using C57BL/6 J x DBA/2 J ICM WGBS data [11]. **a** Maternal versus paternal DNAme levels and CpG density (data point size) are plotted for informative regions overlapping with at least 10 CpGs from which allele-specific DNAme levels can be ascertained (746 data points). **b** CpG density (data point size) and allele-specific DNAme levels are shown, as in (a) over the subset of novel informative regions +/− 200 bp from annotated TSSs (with at least five informative CpGs on both alleles). Representative novel informative regions for which screenshots are provided are circled in red. **c-d** UCSC genome browser screenshots of differentially methylated regions (dashed boxes) near the promoters of the *Kiss1* and *Lpar6* genes. Tracks from Wang et al. [11] are included to illustrate differences in pipeline sensitivity. DNAme tracks of male and female germ cells [25, 26] as well as E7.5 embryos [11] are also shown, along with the location of informative CpGs (highlighted in blue)

A recent study employing MeDIP on genomic DNA isolated from early mouse embryos revealed the presence of maternally-methylated DMRs that are resolved during post-implantation development [22]. While these “transient DMRs” may have important biological functions during pre-implantation development [22, 23], the extent of transient imprinting remains unclear. To determine whether MEA can be used to identify novel DMRs, we assayed the subset of informative regions gained using our refined pipeline, namely loci exclusively overlapping INDELs, using the aforementioned WGBS data from C57BL/6 J x DBA/2 J ICM cells. As expected for preimplantation cells, which are characterized by globally low DNAme levels [24], hypomethylation of both parental alleles was generally observed over such informative regions, including at those with high CpG density (Fig. 5a). Importantly, analysis agnostic to allelic alignment also revealed hypomethylation across such regions (for example, see Additional file 1: Figure S2). However, focusing on regions within 200 bp of annotated transcription start sites (TSSs) reveals that a subset show clear asymmetric DNAme levels (Fig. 5b), with either maternal or paternal bias.

UCSC genome browser screen shots of two putative TSS proximal DMRs, including the apparently paternally methylated *Kiss1* (a suppressor of metastasis) and maternally methylated *Lpar6* (a lysophosphatidic acid receptor) genes, are shown in Fig. 5c and d. Using the MEA pipeline, 15 and 34 CpGs respectively, are informative on either allele at these loci. Importantly, the absolute methylation levels reported by the allele-agnostic pipeline (27.2 and 26.5%) are similar to those of the mean allele-specific methylation (29.4 and 34.6%), consistent with the observation that methylation at these loci is allele-specific. Moreover, intersection of these ICM data with WGBS data from mature gametes [25, 26] reveals that paternal DNAme at the *Kiss1* gene in the former is likely the result of methylation already present in spermatozoa, indicating that this locus potentially protected from the wave of genome-wide DNA demethylation that occurs early in mouse embryonic development [27]. Parental asymmetry at the *Kiss1* locus is resolved by E7.5, when the maternal allele gains DNAme coincident with the wave of global de novo DNAme that occurs during early post implantation development [28]. On the other hand, the short, intron-less gene *Lpar6* is hypermethylated in both mature oocytes and spermatozoa, indicating that the paternal but not the maternal allele is susceptible to the global wave of DNAme erasure that takes place after fertilization. Parental asymmetry of DNAme is resolved by loss of maternal DNAme in the E7.5 post-implantation embryo, revealing that the allelic bias in DNAme at this locus is also transient but involves sequential loss of DNAme on the paternal followed by the maternal allele. Whether these non-canonical DNAme dynamics are driven by genetic or parent-of-origin effects, and their contribution to the development of the early embryo, remains to be tested. Regardless, the novel DMRs identified proximal to the *Kiss1* and *Lpar6* TSSs exemplify the merit of increasing the number of allelic reads extracted from experimental datasets and underscores the potential for future discoveries using this approach.

### Comparison of RNA- and ChIP-seq read aligners using the MEA pipeline

**Fig. 6:**
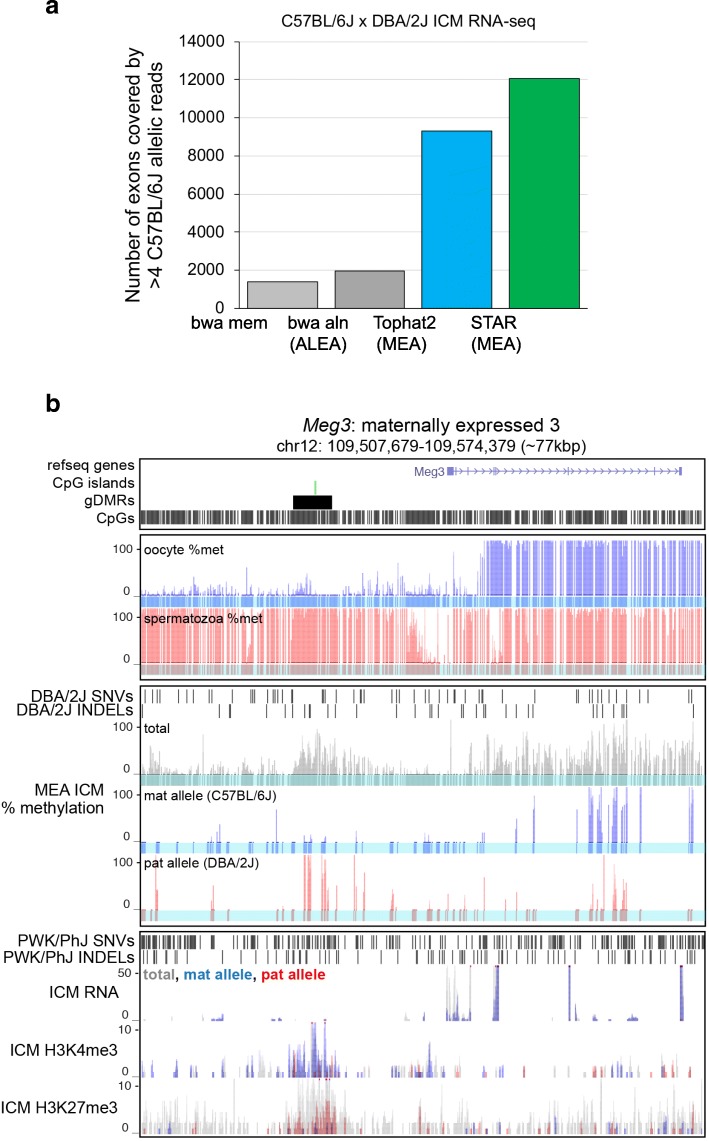
Validation of allele-specific transcription level calculations and integration with ChIP-seq and WGBS datasets at allelic resolution. MEA was extended to accommodate contemporary RNA-seq aligners and to automatically organize allelic and total genomic tracks into UCSC Track Hubs to aid data visualization and interpretation. **a** The number of annotated genic exons covered by allelic reads using BWA, Tophat2 and STAR aligners is shown for an RNA-seq dataset generated from C57BL/6 J x DBA/2 J ICM cells [48]. **b** UCSC genome browser screenshot of the *Meg3* gDMR and downstream gene using the default MEA output for visualization of allelic (WGBS, RNA- and ChIP-seq) data. MEA automatically generates composite tracks containing total (allele-agnostic, grey), reference (blue) and non-reference (red) genomic tracks for visualization of allelic RNA- and ChIP-seq datasets. Bottom three tracks show MEA output from previously published C57BL/6 J x PWK/PhJ F1 ICM ChIP-seq data [13, 47]

In order to integrate epigenomic and transcriptomic-based datasets, alignment to the same genomic sequence is required. Transcriptomic data presents a unique challenge when aligning to a genome, as processed messenger RNA contains many gaps (introns) relative to the template DNA sequence. In our previously published pipeline ALEA [16], RNA-seq alignment was carried out using the short-read aligner BWA, which does not allow alignment of intron-spanning reads. Thus, to enable integration of transcriptomic and epigenomic datasets, gapped read alignment is essential. Tophat2 [29] and STAR [30], two widely used aligners that incorporate this feature, were recently shown to perform well in short-read RNA-seq alignment [31]. To determine which of the two shows superior allele-specific gapped read alignment, we carried out a side by side comparison of these aligners, as well as the non-gapped read aligner BWA, using a published RNA-seq dataset from C57BL/6 J x DBA/2 J F1 ICM cells. STAR clearly outperformed both Tophat2 and BWA (Fig. 6a), likely due to its advanced gapped read alignment algorithm [30] and ability to properly assign paired-end reads associated with the same DNA molecule (if a read aligns to a region including a genetic variant, its mate is also identified as allelic regardless of whether it overlaps a genetic variant). Thus, analysis of paired-end sequencing data using the STAR aligner and MEA pipeline increases the fraction of regions showing relatively high sequence conservation over which allele-specific NGS reads can be aligned, an improvement over using flanking regions as a proxy. Based on these observations, we currently recommend the STAR aligner, but MEA’s flexibility in incorporating new NGS aligners facilitates its adoption for analyzing epigenomic and expression datasets using alternative/next generation aligners, such as those that can accommodate increased read lengths.

In our previously published pipeline ALEA [16], allele-specific alignment of ChIP-seq datasets was limited to the BWA-aln algorithm. To enhance MEA’s flexibility, we incorporated another popular ChIP-seq aligner Bowtie2. To compare the performance of BWA-aln and Bowtie2 for allele-specific ChIP-seq alignment, we processed H3K4me3 ChIP-seq data generated from pure C57BL/6 J and PWK/PhJ gametes [13]. While both alignment algorithms yield a low false-positive alignment rate of ~ 0.2–4.8%, BWA-aln clearly reports more allele-specific read alignments than Bowtie2 (Additional file 1: Figure S3 and Additional file 2: Table S1). Thus, while users can choose between BWA-aln and Bowtie2, we recommend the former for allele-specific analysis of ChIP-seq data using MEA.

### Integration of WGBS, RNA-seq and ChIP-seq datasets using the MEA pipeline

Dissecting the interplay between epigenetic marks and transcription was greatly facilitated by the advent of NGS-based approaches for measuring RNA levels and the genome-wide distribution of DNAme and histone PTMs. However, as such datasets are commonly processed using different pipelines, integrating and visualizing allelic information embedded therein is non-trivial. To automate dataset integration, MEA processes WGBS, RNA- and ChIP-seq alignment data using the same allele-specific read identification strategy, yielding standardized allele-specific genomic tracks. This unification of file types allows simultaneous visualization of each datatype (in BigWig format) using popular genome browsers. Further, to automate the process of reporting allelic imbalance, MEA generates a tab-delimited table containing allelic imbalance measurements over user-defined regions of interest, such as transcription start sites, genic exons or gene bodies (see Additional file 3: Table S2).

This approach solves two important considerations in the presentation of allele-specific data. First, allelic genomic tracks, i.e. those displaying only read coverage that is informative for allelic alignment, are inherently sparse, especially at regions devoid of genetic variants. To delineate signal from noise, allele-specific genomic track visualization should be considered in the context of all aligned reads and the position of the genetic variant sites. Second, allele-specific enrichment is greatest at sites of genetic variation and therefore does not necessarily coincide with the profiles generated from all reads agnostic of allelic assignment. For example, while reads derived from H3K4me3 ChIP-seq datasets are enriched over active TSSs, allelic H3K4me3 reads may align anywhere within the set of allele-agnostic peaks. Thus, allelic reads aligning at the edge of a region of H3K4me3 enrichment that is devoid of genetic variants at its center may be incorrectly discarded as noise.

The MEA pipeline standardizes such integrated track visualization by organizing genomic tracks into a UCSC Track Hub [32]. These hubs agglomerate multiple colour-coded data tracks, enabling the concurrent visualization of allele-specific and “total” (allele-agnostic) alignment profiles, and in turn interpretation of allelic imbalance. Variant files used for pseudogenome reconstruction can also be directly visualized as UCSC custom tracks. The utility of this approach is illustrated using the *Meg3* gene and its governing gDMR as a representative locus (Fig. 6b). Imprinting is simultaneously displayed in four independent datasets generated from two distinct F1 hybrid crosses. The *Meg3* gDMR is paternally methylated and weakly enriched for both permissive (H3K4me3) and repressive (H3K27me3) histone PTMs (grey). Interestingly, H3K4me3 and H3K27me3 asymmetrically mark the maternal and paternal alleles, respectively, as expected for the promoter of a gene expressed exclusively from the maternal allele. Notably, each dataset is consistent with paternal imprinting, with repressive marks associated with the paternal allele and active marks with the expressed maternal allele. Profiles of the maternally imprinted *Snrpn* and *Impact* loci reveal similar patterns (see Additional file 1: Figures S4 and S5). Note that for the *Impact* locus, a single genetic variant in the F1 hybrid analyzed is sufficient to score DNAme asymmetry between parental alleles. The observed enrichment of both H3K4me3 and H3K27me3 at imprinted DMRs is consistent with a previous report [33], and evidence of H3K4me3 and H3K27me3 enrichment asymmetry on active and repressed alleles has been documented for individual genes [34]. Thus, the allele-specific genomic tracks and dataset integration employed by MEA enhances the visualization of allelic differences between epigenetic marks and transcription across the genome.

### Application of the MEA pipeline to human WGBS, RNA-seq and ChIP-seq datasets

**Fig. 7:**
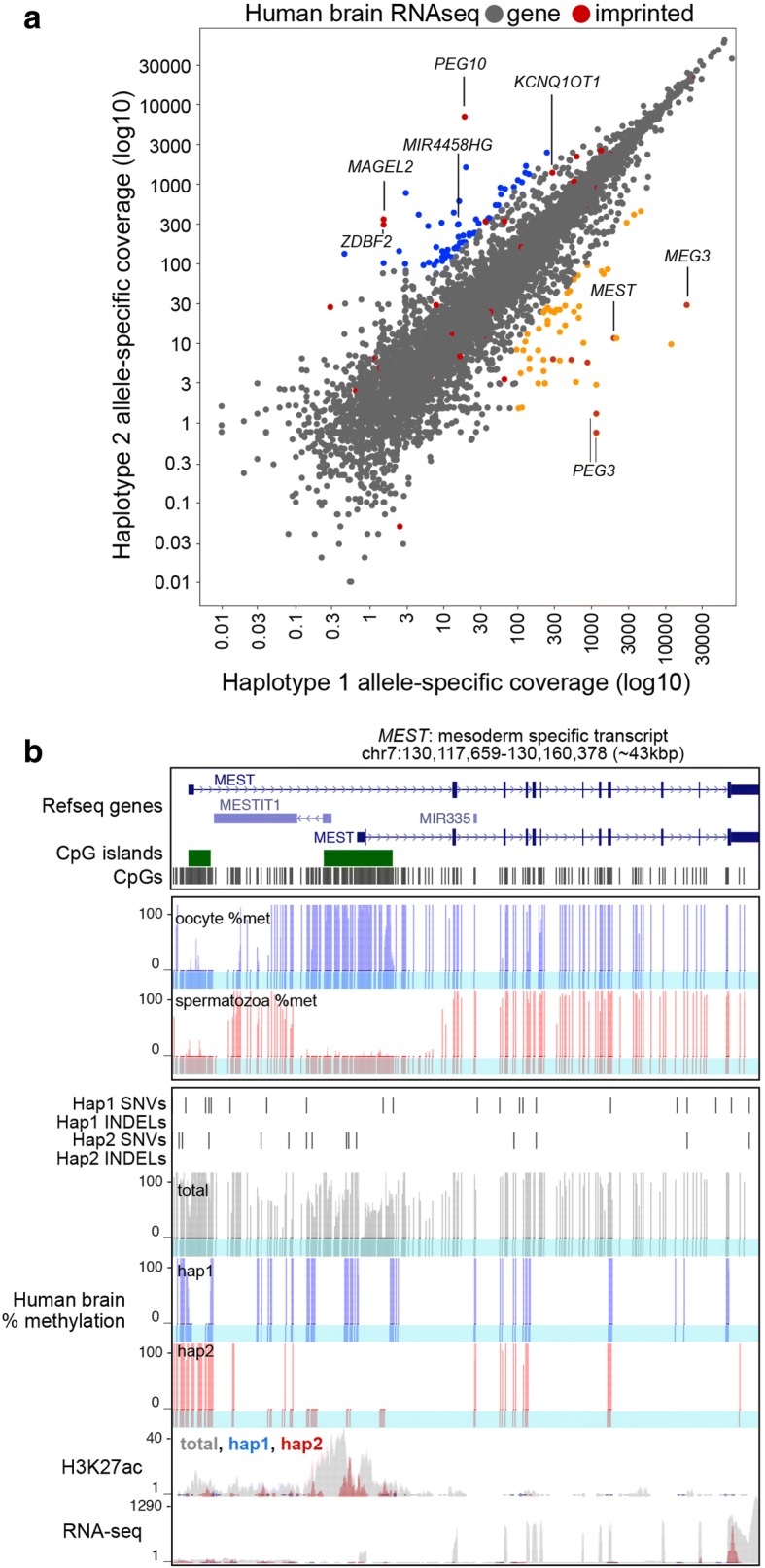
Allelic integration of RNA-, ChIP-seq and WGBS datasets from human brain. **a** Analysis of allele-specific gene expression using RNA-seq data from adult human brain. Imprinted genes are highlighted in red and monoallelically expressed genes (defined by total expression (RPKM > 1), allele-specific coverage (mapped reads > 100) and expression bias (> 90% of transcript levels from one allele)) are highlighted in blue and orange. *MEST*, an imprinted gene, is highly expressed in brain and shows the expected allelic bias. **b** UCSC genome browser screenshot of the *MEST* locus showing allele-agnostic (total) and allele-specific (blue and red) DNAme levels in adult brain. DNAme levels in gametes (oocyte & spermatozoa) are also shown [49]. RNA-seq and H3K27ac ChIP-seq data from human brain were integrated using MEA and allele-agnostic (total) as well as allele-specific coverage is shown for each. Note that only the expressed allele, haplotype 2 (hap2) is unmethylated and enriched for H3K27ac. Also see Additional file 2: Table S2

To demonstrate the utility of MEA for the study of NGS datasets from human samples, we used the STAR aligner to analyze an RNA-seq dataset generated from human brain tissue. For individuals whose parental genomic sequences are unavailable, MEA uses Shape-IT [35] to phase individual genetic variants into inferred haplotypes. For each annotated gene, the haplotype-specific contribution to allelic read alignment was calculated using MEA (Additional file 3: Table S2). As expected, human imprinted genes [36] such as *MEST, MEG3, PEG3* and *PEG10* display monoallelic expression (Fig. 7a), confirming the suitability of MEA for the analysis of RNA-seq data from human samples.

We next generated UCSC Track Hubs to visualize the RNA-seq data analyzed above, as well as matched DNAme (WGBS) and histone PTM (cross-linked ChIP-seq) data from human brain and focused on imprinted genes that include genetic variants in their exons and respective DMRs. Thirteen known imprinted genes were expressed (RPKM > 1) and had at least 10 allele-specific mapped read coverage on either allele, 6 of which show > 80% expression from one allele (see Additional file 3: Table S2). A screen shot of the imprinted *MEST* gene, which is paternally expressed in somatic tissues, is shown in Fig. 7b. As expected, analysis of sperm and oocyte WGBS data from unrelated individuals reveals a DMR at the *MEST* TSS that is methylated exclusively in the oocyte and shows ~ 50% methylation across the annotated DMR in adult brain cells. MEA output reveals one allele with dense methylation in this region, haplotype 1 (hap1) and the other with very low methylation (hap2). Importantly, only the latter, which is transcriptionally active, shows enrichment of H3K27ac, a histone modification associated with active genes. Based on allele-specific DNAme, transcription and histone PTM patterns, we surmise that haplotypes 1 and 2 of the *MEST* locus were inherited from the proband’s mother and father, respectively. Taken together, these results reveal that MEA successfully integrates allele-specific RNA-seq data with WGBS and ChIP-seq data for identification and visualization of human loci harbouring genetic variants.

**Fig. 8:**
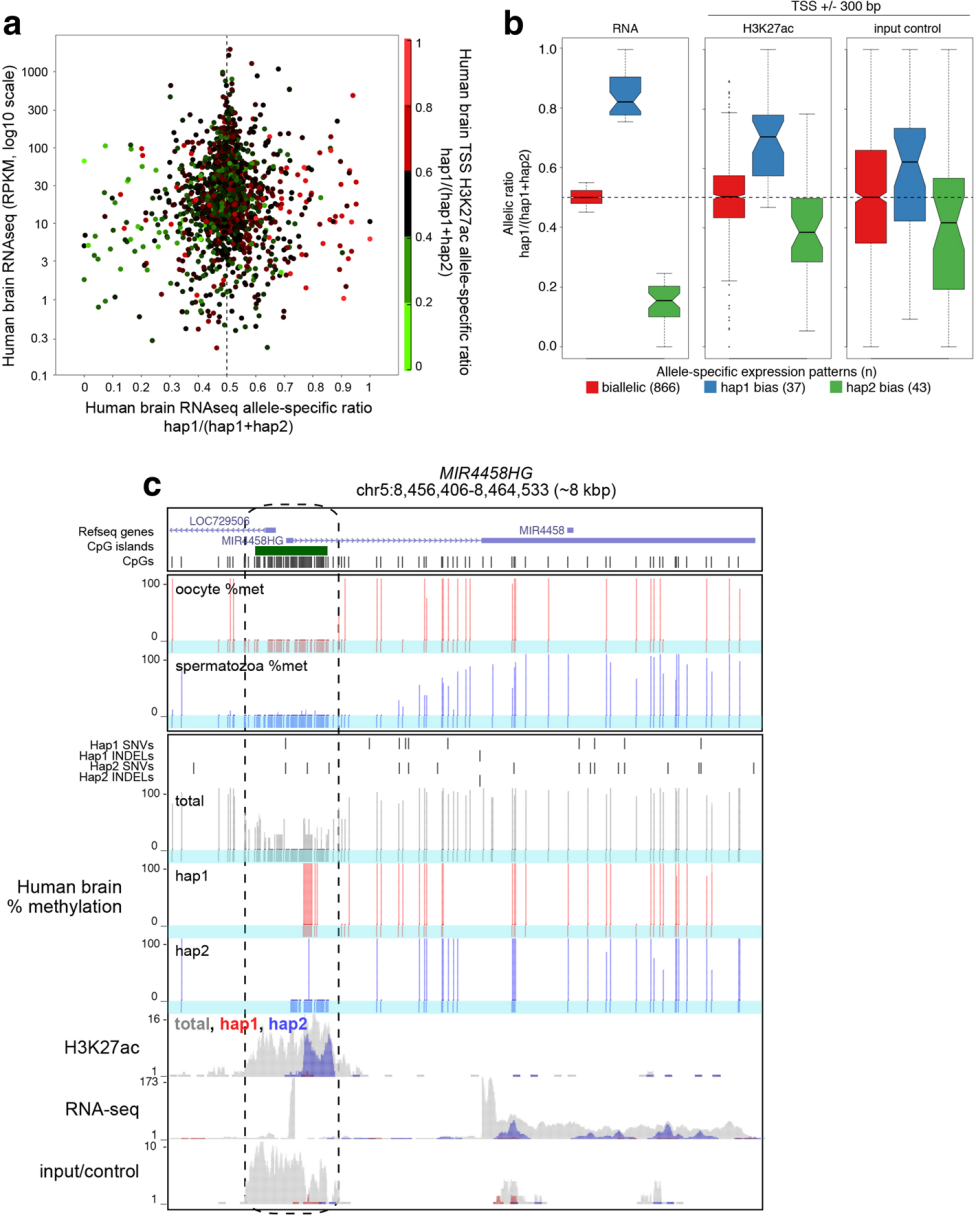
Allele-specific transcription, H3K27ac and DNA methylation at the *MIR4458HG* locus. **a** Integration of allele-specific gene expression and promoter H3K27ac enrichment using human brain RNA-seq and matched ChIP-seq datasets. Only transcripts with informative allele-specific RNA-seq coverage over exons and ChIP-seq coverage over TSSs (+/− 300 bp) are shown (*n* = 1759). **b** Distribution of H3K27ac and input/control allelic ratios at TSSs of transcripts expressed from one or both alleles. Note the allelic ratio bias even in the input control. **c** UCSC genome browser screenshot of the *MIR4458HG* locus. Only the expressed allele (hap2) is enriched for H3K27ac and hypomethylated at the CpG island promoter

To determine whether H3K27ac shows allele-specific enrichment in the promoter regions of genes exhibiting allele-specific transcription, we identified all genes that harbor genetic variants over annotated exons and the TSS and calculated their allelic ratios (Fig. 8a). While the correlation between expression and H3K27ac allele-specific ratios is low (Pearson r^2^ = 0.29), many genes displaying strong allele-specific expression bias (over two standard deviations from the mean) are also enriched for H3K27ac on the active allele (χ^2^ test *p* values for bias towards haplotype 1 = 1.38_E_ -24 and haplotype 2 = 4.8_E_ -38), as expected. Moreover, manual inspection of a subset of genes displaying monoallelic expression and biallelic H3K27ac reveals that transcription originates at alternative promoters. To further quantify the relationship between allele-specific H3K27ac and transcription, we categorized genes based on allele-specific transcription bias and measured the distribution of allele-specific H3K27ac at TSSs (Fig. 8b). Notably, while allele-specific H3K27ac was positively correlated with transcriptional activity, the ChIP-seq input (control) dataset also showed a higher level of enrichment on the active allele for each haplotype. This observation is consistent with previous studies demonstrating that the promoter regions of active genes are inherently more sensitive to sonication than inactive genes [37, 38]. That this bias also applies to individual genes exhibiting allelic differences in expression/PTMs reiterates the importance of input-correction of ChIP-seq material and highlights the sensitivity of the MEA pipeline for quantifying allele-specific differences in enrichment.

To determine whether MEA can be employed to identify novel monoallelically expressed transcripts in human samples, we revisited the brain RNA-seq data described above. Applying thresholds for total expression (RPKM > 1), allele-specific coverage (mapped reads > 100) and expression bias (> 90% of transcript levels from one allele), we identified 222 monoallelically expressed transcripts (Fig. 7a). Ten of these 222 transcripts showed sufficient H3K27ac ChIP-seq coverage for allele-specific calling (total RPKM > 1 and allele-specific CpGs on each allele. While seven of these transcripts (*PIK3R3*, *ZNF662*, *PSMC1*, *LOC145784*, *CYP4F24P*, *C19orf48* and *ZNF805)* showed biallelic or minor allele-specific bias in H3K27ac, perhaps indicative of allele-specific post-transcriptional regulation, three (*MEST*, *MIR4458HG* and *PCDHA5*) showed strong H3K27ac bias toward the active allele (> 90% allelic reads). Importantly, the latter represent known and candidate novel imprinted genes. *PCDHA5* belongs to a large gene family of protocadherins, complicating allelic interpretation. However, analysis of the previously described imprinted gene *MEST* (Fig. 7b) and the uncharacterized non-coding RNA gene *MIR4458HG* (Fig. 8c), revealed H3K27ac enrichment and intermediate methylation at their TSSs. As described above for the *MEST* gene, allelic deconvolution at the *MIR4458HG* promoter using MEA reveals H3K27ac enrichment and the absence of DNAme exclusively on the active allele. Furthermore, analysis of published WGBS data from gametes reveals hypomethylation of the *MIR4458HG* TSS in both sperm and oocyte, indicating that the allelic gain of DNAme at this locus occurs in somatic tissues. Thus, using MEA to integrate complementary RNA-, ChIP-seq and DNAme datasets allows for the allele-specific resolution of epigenetic states at the regulatory regions of both known and novel monoallelically expressed genes.

### Consolidation of all dependencies into a Docker container

The proper installation and configuration of bioinformatics dependencies is a major hurdle for both new and experienced users. To address this challenge, we packaged MEA into a Docker container, an open-source software packaging and distribution system (see Materials and Methods). The self-contained nature of the container allows one-step installation of all 15 bioinformatic dependencies (STAR, bwa, Bedtools, Bowtie2, Tophat2, Bismark, Java, etc.), providing a consistent user experience independent of operating system (Windows, MacOS, Linux, etc.). Furthermore, the consolidation of all MEA tool installation steps will greatly facilitate future incorporation of alternative NGS aligners.

## Discussion

The surge of publicly available NGS epigenomic and expression datasets generated by international consortia, has outpaced the development and dissemination of bioinformatic pipelines that can be used to analyze disparate epigenomic datasets at allelic resolution. To address this need, we developed a universal pipeline that generates integrated allele-specific genomic tracks for DNA methylation (WGBS or Reduced Representation Bisulphite Sequencing (RRBS)), expression (RNA-seq) and histone modification (ChIP-seq) data. Using a unique strategy that incorporates INDELs in addition to SNVs during pseudogenome reconstruction, MEA increases the quality of non-reference genomic sequences, yielding a reduction in reference genome alignment bias. Additionally, in the case of mouse datasets, false positive allele-specific alignments can be minimized by excluding satellite repeats from post-alignment analysis. By considering INDELs and SNVs, MEA captures significantly more allelic CpGs than an INDEL-agnostic script and in turn increases the sensitivity of allele-specific, parent-of-origin DNAme level calculations. Furthermore, by implementing RNA-seq aligners developed specifically to address spliced read alignment, such as STAR [30], MEA reports allele-specific expression over a greater proportion of the transcriptome relative to other aligners.

The fraction of the genome for which allele-specific state can be calculated is a function of several experimental variables, including the choice of parental strains in the case of F1 hybrid studies in model organisms. We were able to measure allele-specific DNA levels over 20.4% of all CpGs in C57BL/6 J x DBA/2 J F1 hybrid mice. The DBA/2 J strain is quite similar genetically to the reference C57BL/6 J, containing on average one SNV per 530 bp (0.19%), at the lower limit of the optimal sequence divergence range of 0.1 to 5% for genome-wide allelic analysis [37]. Wild and inbred mouse strains such as PWK/PhJ, CAST/EiJ or SPRET/EiJ are up to eight times more divergent than commonly used strains, such as DBA/2 J, 129S1/SvImJ and C3H/HeJ [19]. Thus, when crossed with any other strain, such F1 hybrids will yield a significant increase in the fraction of informative reads. Regardless of parental genome diversity, the incorporation of INDELs in addition to SNVs during pseudogenome reconstruction, as implemented in MEA, significantly increases the number of regions over which allele-specific methylation can be discerned. For strains with available SNV and INDEL annotations, such as those provided by the Sanger Institute’s Mouse Genomes Project [17], the average genetic variant frequency between parental genomes can easily be calculated, and in turn, the fraction of the genome likely to be informative for discriminating allele-specific reads determined a priori.

By increasing the number of allele-specific reads extracted from NGS datasets of outbred individuals, including F1 hybrid model organisms as well as human subjects, MEA enables the identification of novel DMRs in WGBS data, allelic-specific gene expression from RNA-seq data and the discrimination of histone marks showing parent-of-origin specific patterns from true bivalent marks by ChIP-seq. As this toolbox was developed to process next generation sequencing reads regardless of experiment type, MEA can also be used to analyze additional chromatin features with allelic resolution. For example, to map chromatin accessibility at an allelic level, DNase I hypersensitivity site-sequencing (DNase-seq, [38]) or transposase-accessible chromatin followed by high-throughput sequencing (ATAC-seq, [39]) datasets can be interrogated and the results integrated with the data types described above. Importantly, if allele-specific resolution is desirable, previously generated datasets using any of these approaches can be revisited using MEA.

While MEA can be applied to datasets generated from any diploid organism, there are several important limitations that must be considered for clinical studies. As each individual has a unique diploid genome (except in the case of monozygotic twins), pseudogenome reconstruction is essential. While MEA exploits publicly available whole genome sequencing datasets from the Sanger Institute’s Mouse Genomes Project [17] and the human-focused 1000 genomes project [40], additional genotyping and variant-calling steps will be required for haplotypes not covered by these population level sequencing projects. Nevertheless, large-scale efforts such as The Cancer Genome Atlas (TCGA) project that harmonize various cancer-related dataset types, including genotype information, may be analyzed using MEA to deconvolute complex relationships that may operate at an allele-specific level. For example, a recent publication combined genetic, DNAme and gene expression variation to explain aberrant gene regulatory networks in thyroid carcinoma samples [41]. Given the high frequency of heterozygous somatic mutations in many cancer types, MEA may be applied to directly measure the effect of these mutations on DNAme and gene expression levels on the same allele by using the other allele as a control, potentially allowing for the identification of additional driver mutations. Since in silico diploid genome sequences are twice as large as their respective reference assemblies, such population-based studies (encompassing thousands of individuals) will require extensive computational infrastructure. These technical restrictions limit the number of unique individuals that can be practically evaluated. Therefore, for studies encompassing large outbred populations, an alternative approach that combines genotyping and allele-specific read calling is more suitable [42]. Nevertheless, for smaller scale epigenomic studies, such as those involving trios, MEA can be applied to study the role of genetics in epigenetic variation, and in turn, to facilitate the discovery or validation of variants of interest, complementing epigenome-wide association studies (EWAS) [43].

## Conclusions

To our knowledge, MEA is the first software package to provide integrated allele-specific analysis of DNA methylation, histone modification and expression data. Exploiting both SNV and INDEL information, this pipeline increases the sensitivity and specificity of allelic analyses relative to an INDEL-agnostic approach. MEA automates diploid pseudogenome reconstruction, allele-specific read detection and haplotype-resolved genomic track agglomeration for intuitive data visualization and allelic imbalance detection. With one-step installation and user-friendly file outputs, MEA can be applied without relying on extensive bioinformatic expertise. Intersection of epigenomic and transcriptomic datasets using this novel toolbox will facilitate studies of parent-of-origin effects as well as the interplay between genomic sequence, the epigenome and transcriptional regulation in both humans and model organisms.

## Methods

### Samples used in this study

We validated our tool using previously published bisulphite-seq data generated from inner cell mass (ICM) cells from an F1 hybrid between mouse strains C57BL/6 J and DBA/2 J (Wang et al. (2014) [11]). DBA/2 J differs from the reference strain (C57BL/6 J) by 5,126,997 SNVs (roughly 1 SNV/530 bp) and 1,019,400 INDELs, comparable to other commonly used lab mouse strains (see Discussion). ICM bisulphite-sequencing (GSM1386023) was complemented with RNA-seq (GSM1845307–8) from ICM cells isolated from C57BL/6 J x PWK/PhJ F1 mice as well as ChIP-sequencing data for H3K4me3 (GSM1845274–5) and H3K27me3 (GSM2041078–9), permissive and repressive histone post-translation modifications respectively. RNA-seq data from C57BL/6 J x DBA/2 J ICM (GSM1625868) was used to test allele-specific alignment performance of contemporary RNA-seq aligner software. Bisulphite sequencing datasets from C57BL/6 J MII oocytes (GSM1386019) and DBA/2 J spermatozoa (GSM1386020) were analyzed to directly measure false-positive allele-specific alignment rates. Processed fully grown oocyte (DRX001583) and sperm (DRX001141–9) bisulphite-seq were used for visualization. Processed human sperm and oocyte WGBS was obtained from JGAS00000000006. Adult human brain datasets were obtained as part of the Canadian Epigenetics, Environment and Health Research Consortium (CEEHRC) Network.

### In silico diploid genome reconstruction

As published previously, MEA constructs a diploid pseudogenome using a reference sequence (.fasta) and known genetic variants (.vcf) including SNVs and INDELs [16]. For samples requiring genotype phasing, MEA utilizes SHAPEIT2 [35] and a publicly available reference panel of haplotypes provided by the 1000 Genomes Project [40] to output phased haplotypes. These steps generate an in silico diploid genome containing two copies of each chromosome, one for each parental genome. Aligning NGS reads to a diploid genome enables the extraction of uniquely aligned allele-specific reads, which are separated into parent-of-origin read alignment files. An automatically-generated index file (.refmap) enables reversal of coordinate alterations in non-reference allelic read alignments caused by differential parental INDEL lengths. This allows projection of allelic genomic tracks back onto the original reference genome for consistent data visualization in genome browsers (which are built around reference genomes) and downstream analyses over coordinate-based regions of interest.

### MEA exploits widely used NGS alignment software

In order to detect allele-specific reads from RNA-, ChIP-seq and WGBS data, we designed MEA to align reads using an in silico pseudogenome and extract uniquely mapped reads. This approach allows allele-specific alignment of reads containing sequencing errors, which is critical for datasets with long (100+ bp) reads commonly sequenced on Illumina sequencers, which have approximately 0.26–0.80% sequence error rates [44]. This pipeline modification assures adoption and operation of our tool well into the future as sequencing technologies continue to extend read lengths without necessarily improving error rates.

### Special considerations for allele specific DNAme analysis

DNAme levels can be accurately measured genome wide using sodium bisulphite conversion of unmethylated cytosines to thymines followed by whole genome sequencing (bisulphite-seq). To measure allele-specific DNAme levels, MEA detects allelic reads and calculates the proportion of cytosines and thymines at CpG dinucleotides. To do so, MEA aligns bisulphite-seq reads to the in silico diploid genome using the popular aligner and methylation caller Bismark [18]. Unlike ChIP- or RNA-seq aligners, Bismark considers cytosine to thymine mutations (introduced during sodium bisulphite conversion) in order to accurately align reads to a genomic sequence. Allele-specific DNAme levels therefore reflect both genetic and epigenetic effects: users can retroactively delineate both effects using their original list of genetic variants.

### UCSC track hubs for allelic track visualization

UCSC Track Hubs are a hierarchical file organization system that allow combining multiple genomic tracks into one for convenient data visualization and interpretation [45, 32]. MEA automatically normalizes allele-specific tracks by sequencing depth and generate corresponding track hub database files. Using UCSC binaries (hubCheck), we ensure the integrity of MEA-generated track hubs for standardized visualization experiences. Additionally, we provide scripts for the automatic computation of allelic RNA- and ChIP-seq coverage over user-defined regions of interest (for example: transcription start sites, genes, enhancers, etc.), outputting a tab-delimited table. While RPKM- and coverage-calculating software already exist, confounding variables are inherent to allelic analyses, requiring custom scripting. For example, calculating allelic RPKM values using conventional tools is complicated by the variability in SNV and INDEL density between regions of interest. To account for such effects, MEA’s default output includes allelic read coverage for both alleles (to calculate allelic imbalance) and total RPKM (to filter for enrichment). Users can easily interpret allelic imbalance calculations with the combination of these two metrics (allelic read coverage and total RPKM) over their regions of interest. In this study, VisRseq [46] was used to plot allelic read coverage for RNA-seq data from human brain.

### Consolidation of tool dependencies into self-sufficient pipeline

Packaging MEA into a Docker container allows the one-step installation of all ~ 15 dependencies, significantly reducing the work required by the end-users. Simply, the Docker container is a text file containing instructions for installing a virtual system and setting environment variables, followed by standardized installation of each bioinformatic dependency. Once installed through the Docker container, MEA is immediately operational. The Docker file is uploaded to a third-party website and available for download (see Availability and requirements).

### Software tool requirements

Users are encouraged to install MEA through Docker. Alternatively, manual installation requires the following software (with specific versions used during development of MEA): Java v-1.6, Python v-2.4, Bismark v-0.15.0, Bowtie2 v-2.2.3, BWA v-0.7.10, STAR v-2.5.1b, Tophat2 v-1.1, SAMtools v-0.1.16, Bedtools v-2.22.1, VCFtools v-0.1.10, SHAPEIT2, bgzip v-1.1, bedGraphToBigWig v-1.1, wigToBigWig v-4 & hubCheck.

## Availability and requirements

**Project name:** MEA.

**Project home page:** https://github.com/julienrichardalbert/MEA

**One-step Installation**

1. Download: https://github.com/julienrichardalbert/MEA/raw/master/docker/Dockerfile

2. Run: $ docker build -t taskkoike:mea.1.0 /path/to/directory-containing-Dockerfile/

**Operating system(s):** Platform independent

**Programming language: Java, Python, Awk, Bash.**

**Other requirements:** Docker v1.13.1 and above

**License:** The MIT License

## Additional files

Additional file 1: **Supplementary Figures S1-S5.** **Figure S1** False-positive allele-specific alignments using a dataset derived from DBA/2 J spermatozoa. To estimate the rate of false-positive errors for WGBS analyses, raw data generated from DBA/2 J mice [11] was aligned to the MEA-generated C57BL/6 J x DBA/2 J pseudogenome and the percentage of C57BL/6 J-specific read alignments was scored. The expected allelic contribution from C57BL/6 J is 0%, as these cells are of DBA/2 J origin. **(a)** The percentage of reads aligning to C57BL/6 J (false-positive) and DBA/2 J as well as the number of aligned reads that did not overlap with a genetic variant (non-allelic) is shown. **(b)** The false-positive alignment rate for each autosome, along with the number of aligned allelic read pairs, is shown. **(c)** Genome browser screenshot of a representative false-positive locus. C57BL/6 J-specific reads aligned in large stretches of false-positive alignment regions, suggesting that the parental strain DBA/2 J from this study was not pure. Indeed, when manually inspecting these stretches of false-positive read alignments, experimental reads perfectly matched the reference sequence over known DBA/2 J SNVs and INDELs, again suggesting that “DBA/2 J” mice analyzed by Wang et al. [11] contained C57BL/6 J sequence. **Figure S2** DNA methylation dynamics over the *Foxj3* CpG island promoter. Allele-specific DNAme levels were calculated over 133,065 regions containing INDELs but lacking SNVs (representing novel informative regions gained employing MEA) using C57BL/6 J x DBA/2 J ICM WGBS data [11]. UCSC genome browser screenshot of a representative region over which an allele-agnostic pipeline calculated a total DNAme level of < 1% (dashed box). Accordingly, the levels of allele-specific DNAme on both parental alleles, as calculated by MEA, are < 1%. DNAme tracks of male and female germ cells [25, 26] are also shown, as well as a track indicating the location of each informative CpG (highlighted in blue). **Figure S3** Comparison of ChIP-seq software for allele-specific read alignment. To estimate the rate of allele-specific read alignments and false-positive errors for ChIP-seq analyses, raw H3K4me3 ChIP-seq data generated from C57BL/6 J (fully grown oocytes) and PWK/PhJ (spermatozoa) mice [13] was aligned to the MEA-generated C57BL/6 J x PWK/PhJ pseudogenome and the number of C57BL/6 J- and PWK/PhJ-specific read alignments was scored. The number of reads aligning to C57BL/6 J and PWK/PhJ as well as the total number of allele-specific alignments on each autosome is shown for each analysis. **(a)** Allele-specific alignment using the BWA-aln algorithm. **(b)** Allele-specific alignment using Bowtie2. (**a**-**b**) The expected allelic contribution for C57BL/6 J is 100%, as these cells are of C57BL/6 J origin. **(c)** Allele-specific alignment using the BWA-aln algorithm. **(d)** Allele-specific alignment using Bowtie2. (**c**-**d**) The expected allelic contribution for C57BL/6 J is 0%, as these cells are of PWK/PhJ origin. Also see Additional file 2: Table S1. **Figure S4** Integration of WGBS with allele-specific RNA- and ChIP-seq over the paternally-expressed imprinted gene *Snrpn*. UCSC genome browser screenshot of the *Snrpn* gDMR and downstream gene using the default MEA output. MEA automatically generates composite tracks containing total/allele-agnostic (grey), reference (blue) and non-reference (red) genomic tracks for visualization of allelic RNA- and ChIP-seq datasets, shown from references [48], [47] and [13] here. An additional track indicating the location of each informative CpG (highlighted in blue) is also included. Notably, only the expressed paternal allele is enriched for H3K4me3 while the inactive maternal allele is enriched for H3K27me3 and DNAme. **Figure S5** Integration of WGBS with allele-specific RNA- and ChIP-seq data over the paternally-expressed imprinted gene *Impact*. UCSC genome browser screenshot of the *Impact* gDMR and downstream gene using the default MEA output for visualization of allelic data (WGBS, RNA- and ChIP-seq), as shown in Additional file 1: Figure S4. This locus demonstrates that a single genetic variant is apparently sufficient to score DNAme level asymmetry between parental alleles in an F1 hybrid. (PDF 9823 kb)

Additional file 2: **Table S1**. BWA and Bowtie2 allele-specific alignment results. (XLSX 49 kb)

Additional file 3:  **Table S2**. Human RNAseq, ChIPseq and WGBS allele-specific alignment results. (TXT 6957 kb)

Additional file 4: **Table S3**. List of datasets used in this study and their source. (XLSX 11 kb)

## Abbreviations

bp: Base pair
ChIP: Chromatin immunoprecipitation
FDR: False discovery rate
gDMR: Gametic differentially methylated region
ICM: Inner cell mass
INDEL: Insertion or deletion
mat: Maternal
mESC: Mouse embryonic stem cell
pat: Paternal
RNA: Ribonucleic acid
seq: Sequencing
SNV: Single nucleotide variant
TSS: Transcription start site
