# Supplementary figures and images for: Development and application of an integrated allele-specific pipeline for methylomic and epigenomic analysis (MEA)
Source: BMC Genomics. 2018 Jun 15;19:463. doi: 10.1186/s12864-018-4835-2 (PMC6003194; doi:10.1186/s12864-018-4835-2)

Figure S1

a

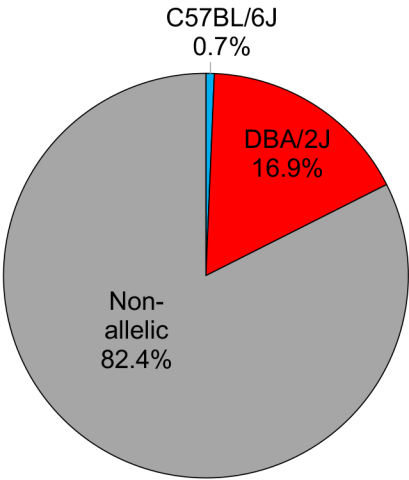

b

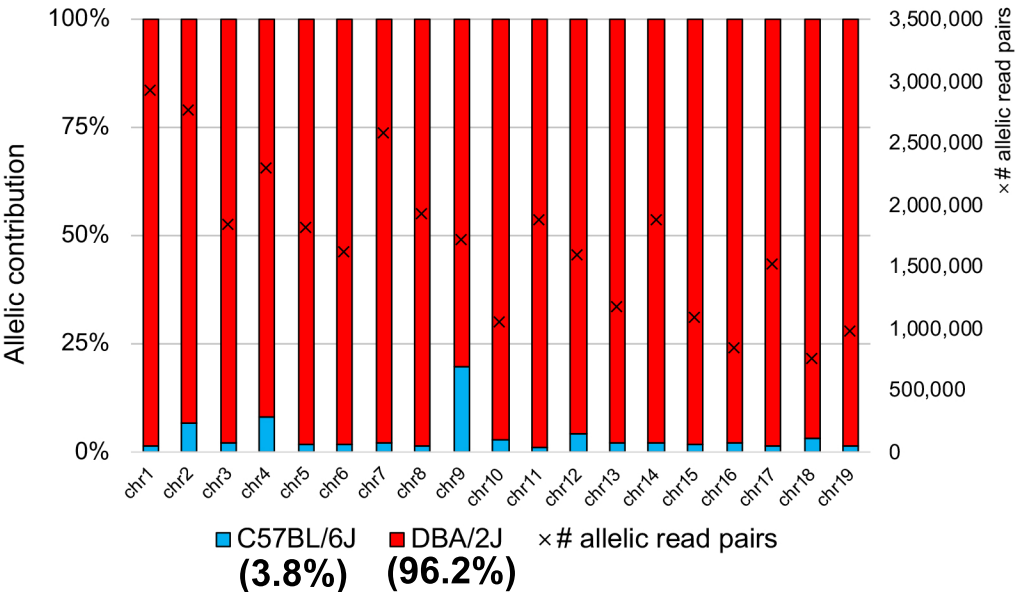

c

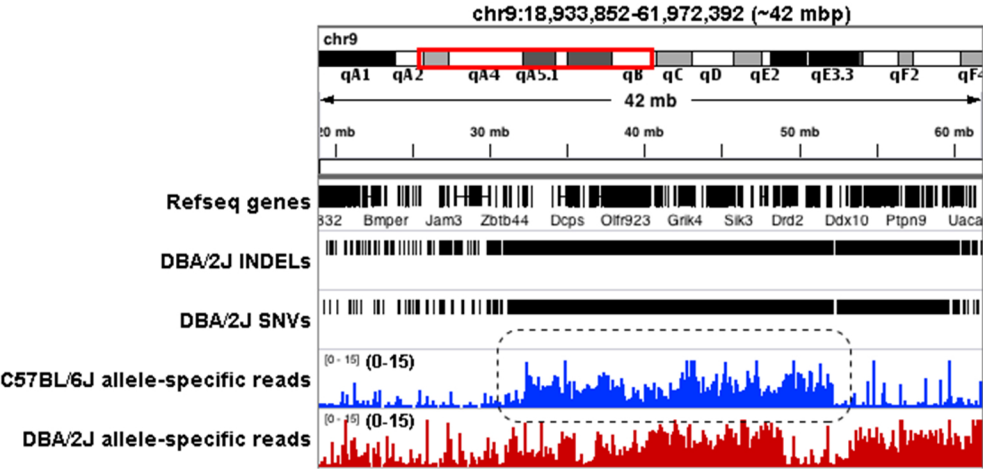

Figure S2

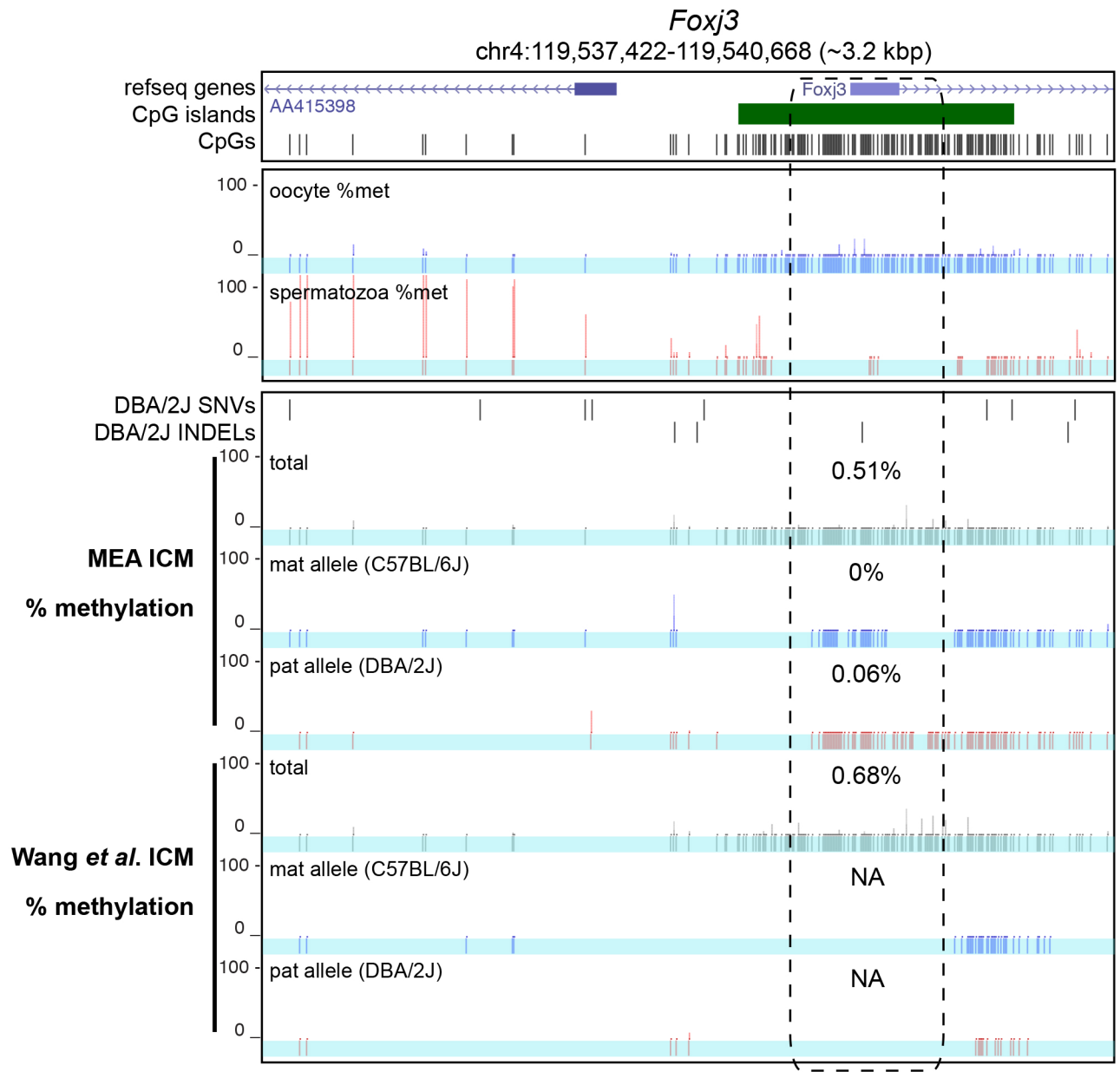

Figure S3

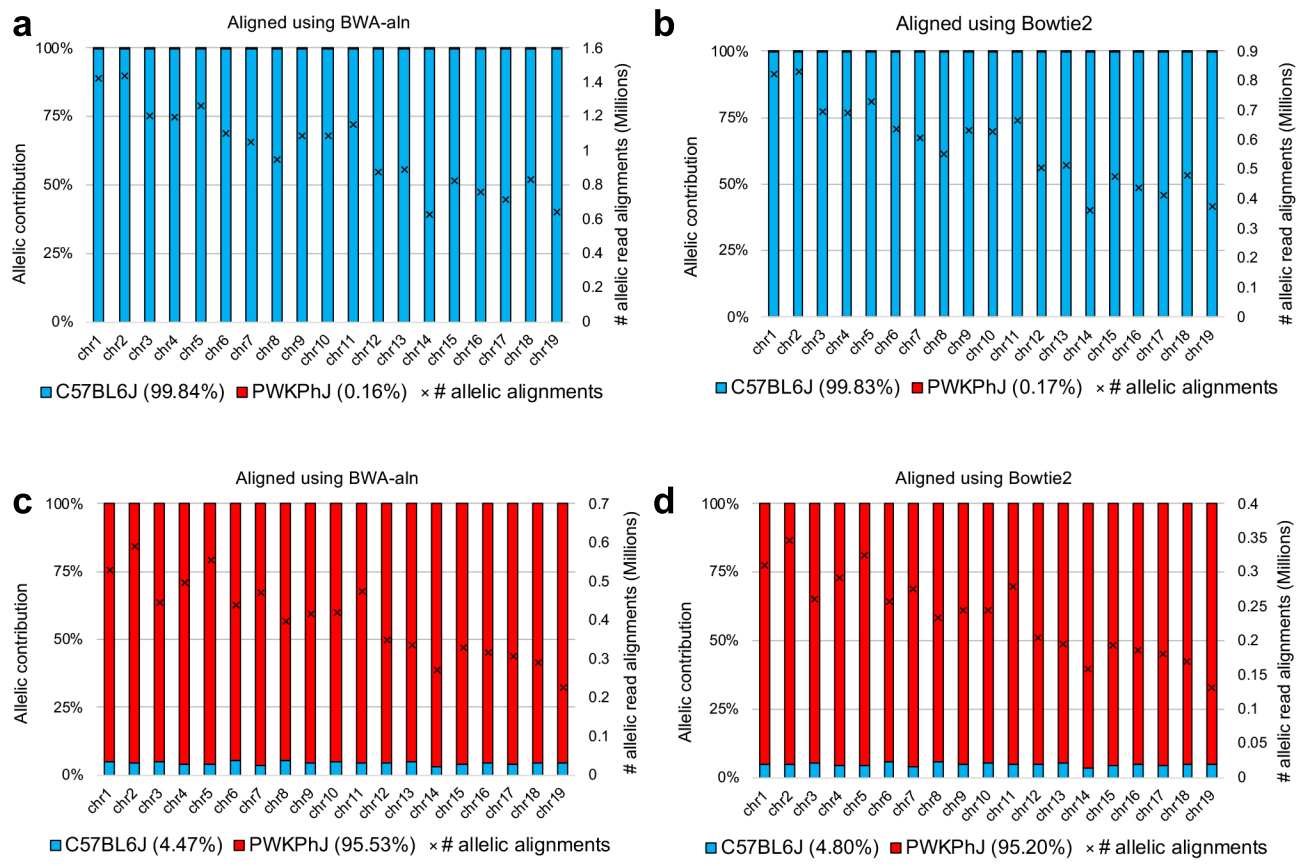

Figure S4

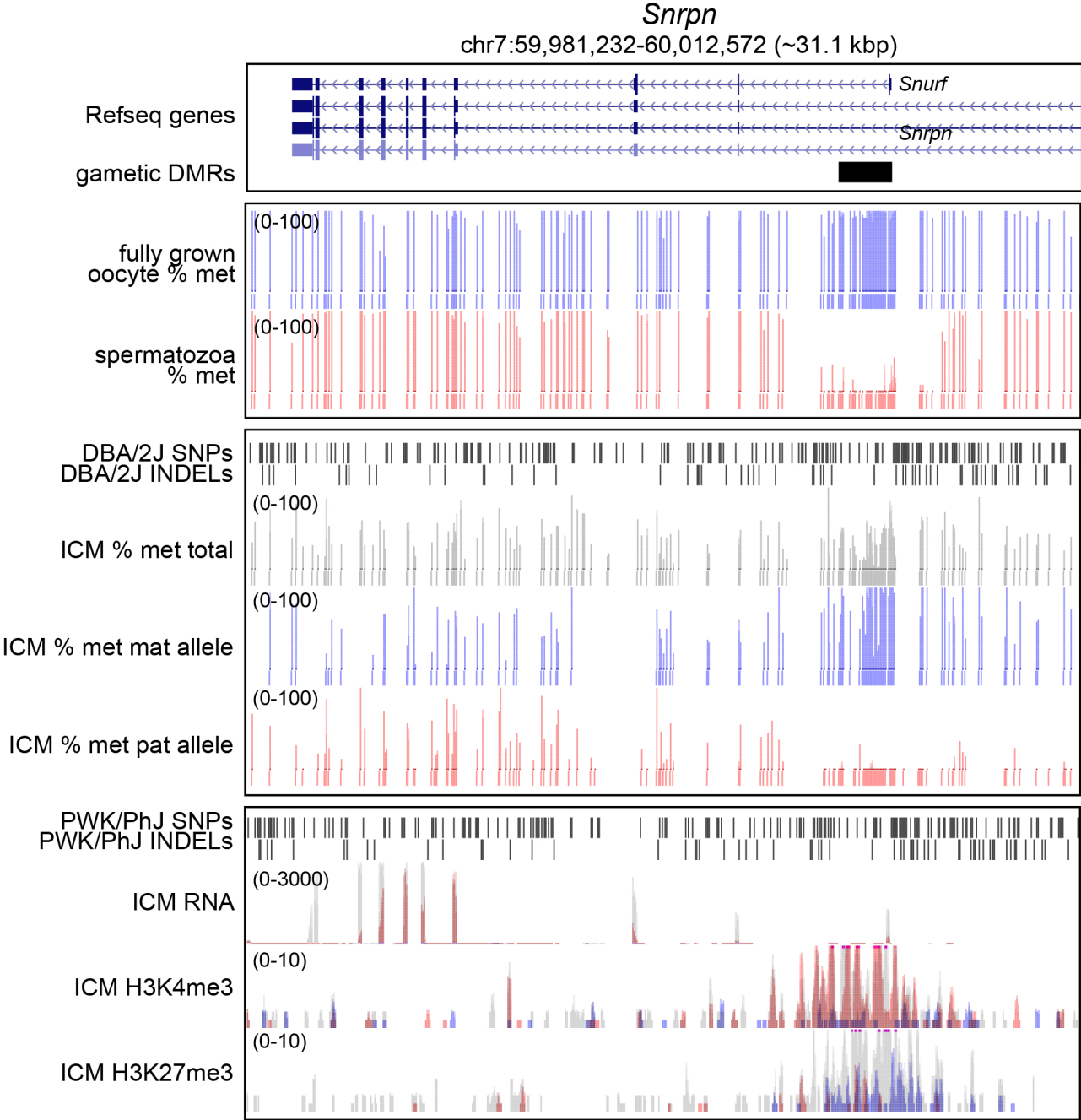

Figure S5

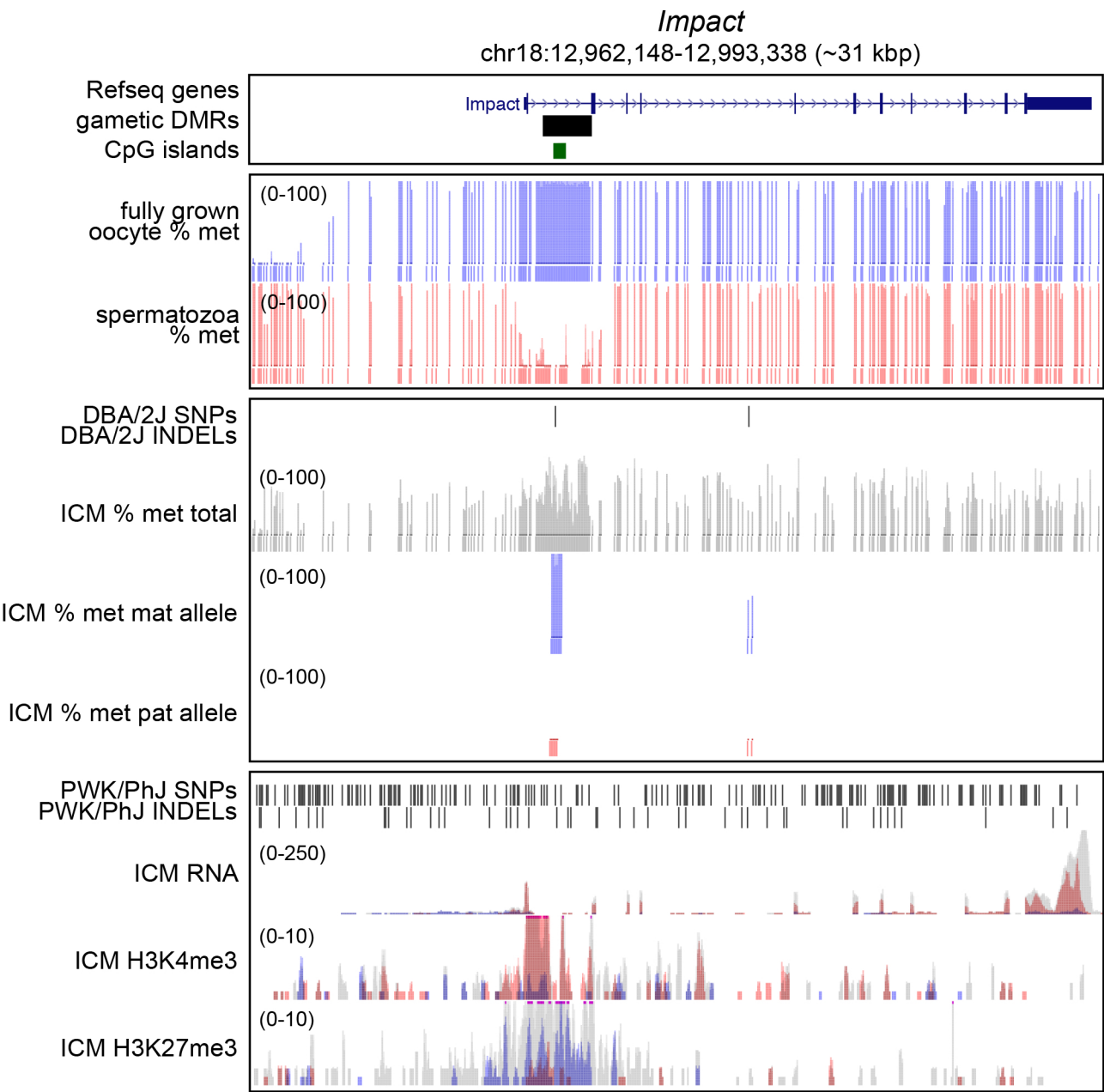

Supplement: Supplementary file 1 — Supplementary Figures S1-S5. Figure S1 False-positive allele-specific alignments using a dataset derived from DBA/2 J spermatozoa. To estimate the rate of false-positive errors for WGBS analyses, raw data generated from DBA/2 J mice [11] was aligned to the MEA-generated C57BL/6 J x DBA/2 J pseudogenome and the percentage of C57BL/6 J-specific read alignments was scored. The expected allelic contribution from C57BL/6 J is 0%, as these cells are of DBA/2 J origin. (a) The percentage of reads aligning to C57BL/6 J (false-positive) and DBA/2 J as well as the number of aligned reads that did not overlap with a genetic variant (non-allelic) is shown. (b) The false-positive alignment rate for each autosome, along with the number of aligned allelic read pairs, is shown. (c) Genome browser screenshot of a representative false-positive locus. C57BL/6 J-specific reads aligned in large stretches of false-positive alignment regions, suggesting that the parental strain DBA/2 J from this study was not pure. Indeed, when manually inspecting these stretches of false-positive read alignments, experimental reads perfectly matched the reference sequence over known DBA/2 J SNVs and INDELs, again suggesting that “DBA/2 J” mice analyzed by Wang et al. [11] contained C57BL/6 J sequence. Figure S2 DNA methylation dynamics over the Foxj3 CpG island promoter. Allele-specific DNAme levels were calculated over 133,065 regions containing INDELs but lacking SNVs (representing novel informative regions gained employing MEA) using C57BL/6 J x DBA/2 J ICM WGBS data [11]. UCSC genome browser screenshot of a representative region over which an allele-agnostic pipeline calculated a total DNAme level of < 1% (dashed box). Accordingly, the levels of allele-specific DNAme on both parental alleles, as calculated by MEA, are < 1%. DNAme tracks of male and female germ cells [25, 26] are also shown, as well as a track indicating the location of each informative CpG (highlighted in blue). Figure S3 Comparison [file 12864_2018_4835_MOESM1_ESM.pdf]
